# The Pathological Mechanisms and Therapeutic Molecular Targets in Arteriovenous Fistula Dysfunction

**DOI:** 10.3390/ijms25179519

**Published:** 2024-09-01

**Authors:** Ruiwei Yan, Anni Song, Chun Zhang

**Affiliations:** Department of Nephrology, Union Hospital, Tongji Medical College, Huazhong University of Science and Technology, Wuhan 430022, China; m202275846@hust.edu.cn (R.Y.); songanni@hust.edu.cn (A.S.)

**Keywords:** arteriovenous fistula, outward remodeling, inward remodeling, vascular smooth muscle cells, extracellular matrix

## Abstract

The number of patients with end-stage renal disease (ESRD) requiring hemodialysis is increasing worldwide. Although arteriovenous fistula (AVF) is the best and most important vascular access (VA) for hemodialysis, its primary maturation failure rate is as high as 60%, which seriously endangers the prognosis of hemodialysis patients. After AVF establishment, the venous outflow tract undergoes hemodynamic changes, which are translated into intracellular signaling pathway cascades, resulting in an outward and inward remodeling of the vessel wall. Outward remodeling refers to the thickening of the vessel wall and the dilation of the lumen to accommodate the high blood flow in the AVF, while inward remodeling is mainly characterized by intimal hyperplasia. More and more studies have shown that the two types of remodeling are closely related in the occurrence and development of, and jointly determining the final fate of, AVF. Therefore, it is essential to investigate the underlying mechanisms involved in outward and inward remodeling for identifying the key targets in alleviating AVF dysfunction. In this review, we summarize the current clinical diagnosis, monitoring, and treatment techniques for AVF dysfunction and discuss the possible pathological mechanisms related to improper outward and inward remodeling in AVF dysfunction, as well as summarize the similarities and differences between the two remodeling types in molecular mechanisms. Finally, the representative therapeutic targets of potential clinical values are summarized.

## 1. Introduction

With aggravated global population aging, chronic kidney disease (CKD) has been a serious public health problem, which ultimately leads to end-stage renal disease (ESRD). It has been well recognized that about 648 per million people globally require renal replacement therapies, including dialysis and transplantation [1]. In the United States, 135,972 new cases of ESRD were reported, with nearly 500,000 patients receiving dialysis and about 250,000 patients receiving kidney transplantation in 2021 [2]. In China, there were about 82 million patients with CKD in 2019, of which 1.47 million (eGFR of <30 mL/min/1.73 m^2^) needed or would soon need renal replacement therapy [3]. Compared with the limited availability and the high cost of donor kidneys, hemodialysis is more appropriate for the majority of ESRD patients. As the core element of hemodialysis, vascular access (VA) mainly includes central venous catheter (CVC), arteriovenous graft (AVG), and arteriovenous fistula (AVF). Among them, AVF is regarded as the best and most crucial VA due to its higher patency rate and lower infection and mortality rates [4,5,6].

As the lifeline of ESRD patients, AVF mainly includes the forearm (radial-cephalic) AVF and upper arm (brachial-cephalic) AVF nowadays. Actually, the first radial AVF was just created in 1966 by Brescia et al., making AVF the most widely accepted VA at present [7,8]. However, the rate of primary unassisted AVF patency at 1 year has been reported to be only approximately 40% [9], which is responsible for great economic burdens and poor life quality [10]. Nowadays, growing evidence has shown that AVF maturation malfunction characterized by outward remodeling failure and AVF stenosis presented pathologically as excessive inward remodeling contribute to AVF dysfunction.

In this review, we summarize the updated knowledge regarding the pathological mechanisms and therapeutic molecular targets in AVF dysfunction. We will discuss the underlying mechanisms of AVF failure involved in two improper vascular remodelings, outward remodeling and inward remodeling, and compare their similarities and differences, as well as display the critical signaling pathways and molecular targets for the promising prevention and treatment of AVF dysfunction.

## 2. The Clinical Characteristics of AVF Dysfunction

In Kidney Disease Outcomes Quality Initiative (KDOQI) 2019, arteriovenous access (AVF or AVG) flow dysfunction refers to clinically significant abnormalities in arteriovenous access flow or patency due to underlying stenosis, thrombosis, or related pathological defects, while AVF functional maturity is defined as the ability to provide a prescribed dialysis consistently with two needles for more than two-thirds of the dialysis sessions within 4 consecutive weeks, as well as ensuring that vessel diameter > 5 mm and blood flow > 500 mL/min are satisfied [11]. According to KDOQI 2019, the assessment of AVF function mainly depends on corresponding physical examinations, including the inspection of the arm, chest, neck, and face and the palpation of the entire AVF tract, arm elevation, pulse, and thrill abnormalities, as well as pulse augmentation tests [11]. In addition, specialized instruments including the ultrasonic dilution method and doppler ultrasound are adopted to detect AVF access flow and anatomical abnormalities [12,13]. Practically, it has been an important indicator for clinical physicians to assess AVF function whether the access is easy to be repeated cannulated or available for adequate hemodialysis. To date, AVF dysfunction repair is mainly divided into post-stenosis and post-thrombotic repair clinically based on corresponding pathophysiological features [11]. Nowadays, balloon angioplasty (with high pressure as needed) is still the first choice for post-stenosis repair, while paclitaxel-coated balloon angioplasty and cutting balloon angioplasty have been gradually developed. In addition to balloon angioplasty, laminar stent also provides an obvious curative effect for AVF stenosis. As for post-thrombotic repair, thrombectomy is the predominant procedure recommended by KDOQI 2019, and early thrombectomy is always associated with better long-term outcomes [14].

## 3. The Pathophysiological Features of AVF Dysfunction

Physiologically, it is well recognized that the venous wall is a three-layer construction including an outer membrane consisting of extracellular matrix (ECM), fibroblasts, myofibroblasts, and inflammatory cells; a middle membrane characterized by vascular smooth muscle cells (VSMCs) and extracellular fibers; and intima mainly composed of endothelial cells (ECs) and a thin basement membrane. Additionally, an inner and outer elastic membrane help to separate three layers of the vascular membrane [15,16]. 

At present, AVF anastomosis can be performed via side-to-side or side-to-end manners between different arteries and veins separately [17]. Among them, the side-to-end AVF between radial artery and cephalic vein is still the most widely used anastomosis [18,19,20]. Once AVF is established, the venous outflow tract exposed to high pressure and shear stress as well as oxygen-adequate environments gradually achieves outward remodeling including wall thickening and luminal dilatation through VSMC proliferation and ECM degradation, deposit, and rearrangement, as well as immunoregulation, ultimately leading to venous arterialization, resulting in blood flow increase, named AVF maturation [21,22]. In turn, AVF dysfunction is attributed to the failure of venous outflow tract outward remodeling at least in part. 

On the other hand, neointimal hyperplasia, also called excessive inward remodeling, has also been reported to contribute to AVF dysfunction to some extent. In response to various insults in AVF status such as surgical injury, hemodynamic disturbance, and mechanical stress, different types of cells including VSMCs, fibroblasts, myofibroblasts, and bone-marrow-derived cells are recruited to the intima, leading to ECM accumulation and inflammation infiltration, as well as cell–cell interaction, ultimately resulting in excessive inward remodeling and AVF stenosis [23]. In the past years, most studies focused on neointimal hyperplasia-induced AVF dysfunction; however, recent studies have also shown that venous outflow tract with untreated intimal hyperplasia stimulated by uremia toxin could also provide adequate blood flow after AVF establishment, ultimately satisfying the need for hemodialysis [24]. Thus, more and more investigators placed a great perspective on the vital role of outward remodeling failure-induced AVF dysfunction. It is hypothesized that AVF dysfunction is attributed to the combined occurrence of outward remodeling failure and excessive inward remodeling.

## 4. The Pathological Mechanisms Underlying Outward Remodeling Failure

**Figure 1:**
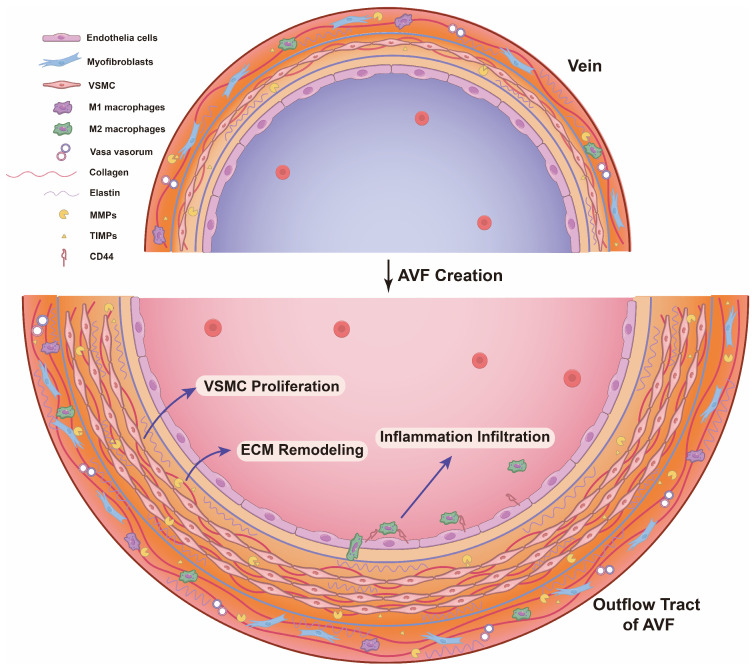
Outward remodeling after AVF creation. The vessel wall is comprised of three layers including the tunica intima, media, and adventitia. Prior to the creation of AVF, there is a limited number of VSMCs in the tunica media and a low ratio of collagen and elastin. Following AVF establishment, hemodynamic changes stimulate the proliferation of VSMCs in the outflow tract of media. Simultaneously, CD44 on endothelial cell surfaces promotes accumulation and infiltration of M2 macrophages, regulating an anti-proinflammatory and anti-inflammatory microenvironment balance within the vascular wall. In addition, myofibroblasts, macrophages, and VSMCs secrete MMPs and TIMPs, which promotes ECM deposition and remodeling and vessel wall thickening, leading to outward remodeling of the AVF ultimately.

Outward remodeling refers to the thickening of the vessel wall and the dilation of the lumen to accommodate the high blood flow in the AVF. Growing evidence suggests that differentiated VSMC proliferation, ECM degradation, deposit, and rearrangement, and inflammation infiltration are involved in outward remodeling. Thus, inadequate abovementioned cellular events contribute to outward remodeling failure (Figure 1).

### 4.1. Differentiated VSMC Proliferation

As a cardinal component of the vascular wall integrality and tension, VSMCs, especially the phenotypic transformation of VSMCs, have been considered as a hot topic in various vascular diseases such as hypertension, atherosclerosis, and arterial calcification. Based on different functions and phenotypes, VSMCs can be classically divided into two phenotypes: (i) differentiated VSMCs labeled with actin-binding protein smooth muscle 22α (SM22α)^+^ [25,26,27], α-smooth muscle actin (α-SMA)^+^ [28,29], myosin heavy chain 11 (SM-MHC/MYH11)^+^ [30,31,32,33], h1-calponin^+^ [34], and smoothelin^+^ [35] are mainly responsible for contractility; (ii) dedifferentiated VSMCs labeled with cytokeratins-8 and 18 (CK-8, CK-18)^+^ [36,37], intercellular adhesion molecule-1 (ICAM-1)^+^ [38], vascular cell adhesion molecule-1 (VCAM-1)^+^ [39] acquire the ability to synthesize and secrete ECM, as well as proliferate. 

In a recent study using a novel VSMC lineage tracing reporter mouse in the AVF model, investigators observed the thickened media in venous outflow tract at 4 weeks after AVF establishment, while VSMCs in the thickened media were exclusively composed of MYH11^+^/Ki67^−^ differentiated VSMCs. Additionally, human specimen analysis results were consistent with the above animal conclusion [40]. Subsequently, Hu et al. found that the media that VSMCs proliferated by activating the transforming growth factor-beta (TGF-β)/TGF-β–activated kinase 1 (TAK1) signaling pathway in human and mouse mature AVFs, regulating collagen 1 and fibronectin expressions, ultimately mediated the vessel wall thickening and promoted outward remodeling [41]. Consistently, another experiment concerning the outward remodeling of inflammation regulation also found that the thickening of vessel walls was due to the proliferation of medial VSMCs [42]. Additionally, VSMCs in venous outflow tracts in RP105^−/−^ AVF mice differentiated towards a regenerative phenotype with reduced proliferation, which eventually resulted in impaired outward remodeling [43]. Thus, differentiated VSMC proliferation plays a vital role in outward remodeling during AVF maturation, which will also be a promising target in prevention and treatment for AVF dysfunction attributed by outward remodeling failure.

### 4.2. ECM Degradation, Deposit, and Rearrangement

As a non-cellular, three-dimensional macromolecular network composed of a well-organized arrangement of various glycoproteins, including collagen, elastin, proteoglycans, glycosaminoglycan, fibronectin, and laminin, ECM is essential for vascular function dependent on its mechanical strength to maintain vascular structure and correlation with cell components to regulate their phenotypes [44]. ECM deposit in vascular walls relies on the balance between synthesis mediated by different cells such as VSMCs, myofibroblasts, and fibroblasts and degradation induced by the disturbance between matrix metalloproteinases (MMPs), members of a zinc-dependent proteases family, and corresponding tissue inhibitors of metalloproteinases (TIMPs) [45]. Growing evidences has provided a comprehensive review regarding the dual role of ECM in outward remodeling in AVF maturation.

On the one hand, accumulating data have showed that ECM degradation induced by the activation of MMPs and the inhibition of TIMPs contributed to the maturation of AVF. As reported by a study enrolling 20 patients treated with AVF surgeries including 13 mature and 7 failed, serum MMP/TIMP levels in patients with mature AVF were significantly higher than those with failed AVF [46]. Meanwhile, it was also found that the upregulation of MMP-2 and MMP-9 kept race with the increase in wall thickness and lumen diameter in AVF rats [47]. As an important component of ECM degraded by MMPs, elastin has been reported to be involved in the pathogenesis of aortic aneurysms and uterine artery remodeling during pregnancy [48,49,50]. Consistently, it has been noted that elastin haplodeficient (eln^+/−^) mice with AVF surgeries obtained a larger lumen area compared to controls, suggesting that the lower abundance of elastin led to enhanced outward remodeling [51]. It is well recognized that Relaxin (RLN) could act on the vascular system by interacting with RLN/insulin-like peptide family receptor 1 (RXFP1), leading to vasodilation, ECM deposit, and reduced inflammation. As reported, Rxfp1^−/−^ mice presented a 22% reduction in venous outflow tract size 14 days after AVF surgeries, accompanied by a 43% increase in elastin abundance and a 41% decrease in elastase activity [52]. Nowadays, a phase III clinical trial focusing on outward remodeling-mediated AVF maturation by promoting elastic fiber degradation has been conducted successfully [53]. 

Interestingly, it has been hypothesized that AVF maturation is also attributed to increased ECM accumulation to some extent. In murine AVFs, investigators found that the expressions of MMP2 and MMP9 gradually decreased to the baseline levels 7 days after AVF establishment, accompanied by increases in collagen, elastin, and other ECM components [54]. Simultaneously, high levels of MMP expression often predict a persistent state of inflammation and lead to AVF maturation failure. It has been reported that the AVF maturation rate was significantly improved after natural vascular scaffolding (NVS) therapy in rats with femoral AVF accompanied by the decreased expressions of MMP-2/9 and inflammatory marker IL-6, suggesting a negative correlation between MMP-2/9 and AVF maturation [55].

ECM rearrangement also exerts a complicated influence on AVF maturation in addition to ECM degradation and deposit. In this case, the secretion of glycoproteins and non-collagenous proteins is a key cellular event in outward remodeling, contributing to a thorough maturation of AVF [54]. In a study of bulk RNA sequencing (RNA-seq) that collected veins and AVFs from 38 patients with ESRD, it was found that the spatial arrangement of ECM components was also further perfect in AVFs and had an important impact on the functions of AVFs [56]. In healthy vessels, the media are highly rich in elastin and collagen fibers, organized in concentric structural units, and can be extended and straightened in the circumferential direction [16]. Pathologically, the circumferential orientation of postoperative perivascular lumen fibers has been reported to be associated with AVF immaturity in patients undergoing AVF surgeries. Meanwhile, new collagen fibers are always deposited in an almost wholly pre-stretched form, thus preventing any further dilation to allow outward remodeling [6]. In keeping with above studies, after treatment with NVS therapy in rat AVF in another animal experiment, collagen fibers in the vascular wall trended toward perpendicular alignment concerning the lumen circumference in the NVS-treated AVFs and acquired a better maturation rate of the AVF towards perpendicular remodeling [55]. 

Taken together, ECM degradation, deposition, and rearrangement are exquisitely regulated during AVF maturation through multiple mechanisms. Determining the time point of key molecular events and preventing excessive degradation or deposition in ECM regulation will be beneficial to the treatment of AVF dysfunction induced by outward remodeling failure.

### 4.3. Inflammation Infiltration

Inflammation infiltration is a pivotal pathophysiological event throughout the whole process from AVF establishment to maturation or failure, and it largely determines the microenvironment of AVF, which in turn affects the phenotypic transformation of VSMCs, ECM remodeling, and the outcome of AVF [43,57,58]. At the beginning of AVF establishment, ECs are firstly exposed to shear stress and secrete large amounts of cytokines including platelet-derived growth factor (PDGF) [59]. PDGF induces monocytes differentiated into macrophages, producing various pro-inflammatory mediators such as tumor necrosis factor-alpha (TNF-α), monocyte chemotactic protein-1 (MCP-1), and interleukin-8 (IL-8) [60,61], which activates ECs to ICAM and VCAM in turn, ultimately leading to leukocytes binding with ECs and a pro-inflammatory microenvironment. Similar to ECM remodeling, inflammation infiltration in AVF maturation requires a balance between pro-inflammatory and pro-resolving signaling to promote outward remodeling, where sustained pro-inflammatory microenvironment leads to adverse events such as the proliferation and migration of VSMCs into the intima and the excessive deposition of ECM and lipid, eventually resulting in AVF dysfunction [62].

Among the different kinds of immune cells, macrophages, consisting of two phenotypes named as M1 (pro-inflammatory) macrophage and M2 (anti-inflammatory) macrophage, respectively, have been the hottest topic. It is well recognized that M1 macrophages predominate in the early stage of inflammation by secreting numerous pro-inflammatory cytokines and providing an inflammatory microenvironment, whereas M2 macrophages predominate in the late stage of inflammation through producing anti-inflammatory mediators and resolving inflammation response [58]. Guo et al. demonstrated that the inhibition of M1 macrophages with rapamycin in the early phase was beneficial to outward remodeling in the AVF mouse model [63]. However, the depletion of macrophages with clodronate-containing liposomes resulted in reduced vessel wall thickness and reduced patency, suggesting that M2 macrophages may play a beneficial role in outward remodeling [63]. CD44 is a widely expressed cell adhesion molecule and a major receptor for ECM components. During AVF maturation, CD44 promotes M2 macrophages accumulation, ECM deposition, vascular wall thickening, eventually facilitating the outward remodeling of AVF, which may be mediated by MCP-1 [64]. Furthermore, macrophages also could secrete large amounts of MMPs, suggesting that they may play an important role in ECM dynamics and regulate AVF outward remodeling [65].

Additionally, T cells are also reported to mediate AVF maturation by regulating macrophage phenotype. In the mouse aortocaval fistula model, the administration of an anti-programmed death ligand 1(PD-L1) antibody increased T-helper type 1 cells and T-helper type 2 cells but decreased regulatory T cells, resulting in an increase in M1 macrophages and a decrease in M2 macrophages, which were associated with reduced vascular wall thickening and decreased AVF patency [66]. However, a histopathological inspection of AVF from athymic rats showed that T-cell immunodeficiency negatively affected venous vascular remodeling, resulting in reduced lumen and decreased AVF patency [67]. Therefore, more studies are needed to clarify the role of T cells in AVF.

In conclusion, inflammation infiltration is an indispensable cellular event for AVF maturation, especially macrophages and T cells [64,68]. Recently, more and more studies have focused on the role of diverse macrophages and T cells in the outward remodeling of AVF maturation, but we need to pay great efforts to explore the nature of inflammation-related AVF dysfunction.

## 5. The Potential Mechanisms Underlying Excessive Inward Remodeling

**Figure 2:**
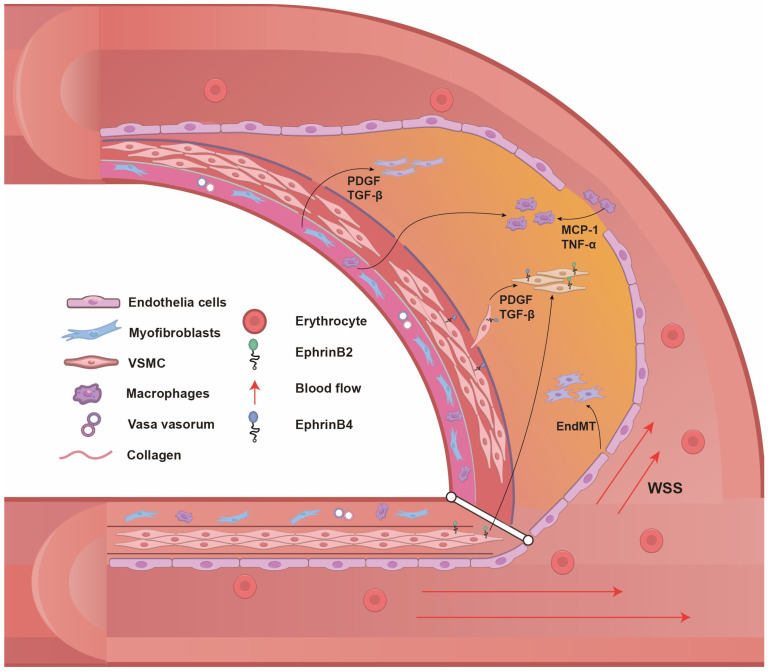
Cellular origin of neointima in AVF. After AVF surgery, endothelial cells undergo endothelial mesenchymal transition (EndMT) by the stimulation of low wall shear stress (WSS) and high toxin environment, which participates in the formation of neointima. At the same time, under WSS stimulation, endothelial cells secrete large amounts of cytokines including PDGF and TGF-β, which activate the dedifferentiation, migration, and proliferation of VSMCs derived from arteries and veins and secrete collagen in the neointima. Endothelial cells upregulate selectin and intercellular adhesion molecules, recruit monocytes into the neointima, and further release inflammatory molecules such as PDGF and TGF-β. Myofibroblasts are activated and migrate to the neointima under the stimulation of PDGF, TGF-β, and other cytokines and secrete a large amount of ECM, which leads to AVF stenosis.

Excessive inward remodeling, characterized by venous neointimal hyperplasia, is a vital pathophysiological culprit leading to AVF malfunction. Growing evidence suggests that ECs, VSMCs, myofibroblasts, fibroblasts, inflammatory cells, and bone-marrow-derived cells altogether participate in neointimal formation (Figure 2).

### 5.1. Endothelia Cells

Following AVF establishment, ECs, as the cell type that directly senses changes in hemodynamics, release diverse mediators that regulate vascular tension, cell growth, platelet function, and blood coagulation and exert a bidirectional effect in AVF stenosis. On the one hand, nitric oxide (NO) derived from endothelial nitric oxide synthase (eNOS) has been recognized as beneficial for vascular health and function [69,70], which could stimulate arterial and venous vasorelaxation [71,72] by increasing the production of cyclic guanosine monophosphate (cGMP), activating smooth muscle relaxation and inhibiting arterial SMC migration and proliferation [73]. In the AVF mouse model with end-to-side anastomosis between jugular vein and carotid artery, eNOS overexpression induced a larger lumen area and a thinner radial wall in venous outflow tract [74]. Jumonji domain-containing protein-3 (JMJD3) is a histone H3 lysine 27 (H3K27) demethylase that promotes endothelial regeneration [75,76]. Recent study observed the knockdown of endothelial JMJD3-reduced eNOS expression and NO production, which resulted in VSMC proliferation and accelerated neointimal hyperplasia in AVF [76]. 

On the other hand, ECs could secrete various factors such as TGF-β, PDGF, IL-1, vascular endothelial growth factor (VEGF), endothelin-1 (ET-1), selectin, and ICAM under shear stress in AVF anastomotic stoma [77], which recruit inflammatory cells to the outflow tract and induce SMCs and fibroblasts that proliferate and migrate to AVF, contributing to neointimal formation [59,62]. At the same time, AVF creation also increased the activation and permeability of ECs, which is conducive to inflammatory cell infiltration in AVF [78]. Moreover, it has been demonstrated that shear stress triggers the proliferation and migration of VSMCs via the extracellular-signal-related kinase 1 and 2 (ERK1/2) signal-transduction pathway in an EC-dependent manner [79,80]. Furthermore, Avastin, a humanized monoclonal antibody against VEGF-A, could prevent venous wall thickening and intimal neointima formation in the proximal outflow vein [81]. Recent studies also have revealed that EndMT is a potential mechanism underlying neointimal hyperplasia in various vascular diseases [82,83]. Chang et al. found a mixed ultrastructural phenotype of ECs and VSMCs under low wall shear stress in luminal ECs of the aortocaval fistula of rats, indicating the presence of EndMT in neointimal hyperplasia [84]. Subsequently, further research by Cdh5-Cre/ERT2;ROSA26-tdTomato transgenic mice for endothelia lineage tracing identified that EndMT was indeed involved in the neointimal hyperplasia of AVF and that the endothelial-derived cells contributed to 24% of the neointimal cells [84]. Similar results were obtained in another study that activated β-catenin signaling in AVF-induced ECs transitioning into myofibroblasts [85]. Wang et al. found abundant mesenchymal markers (fibroblast-specific protein 1 and α-SMA) and activated Notch signaling pathway in the ECs of uremic mice or patients with AVF, ultimately contributing to neointima formation, which could be inhibited by rapamycin partially [86,87].

Thus, ECs play a complicated role in the neointimal hyperplasia of AVF stenosis. It is a potential therapeutic strategy for excessive inward remodeling-induced AVF dysfunction to maintain the ECs’ physical function and inhibit phenotype transformation. 

### 5.2. Vascular Smooth Muscle Cells

It is well recognized that VSMCs located in anastomotic stoma contribute to neointima formation in AVF stenosis [88,89]. Notably, in a Wnt1-cre-GFP mouse model (with only carotid SMCs labeled by GFP) that used an end-to-end connection between the common carotid artery and the jugular vein, researchers found that about half of the cells in the neointima were GFP-positive, confirming that arterial VSMC migration was also involved in neointima formation through the Notch1/RBP-Jκ signaling pathway [89]. Under physiological conditions, the most significant difference between the arterial and venous wall lies in the tunica media. Arterial tunica media is thicker, and there are more layers of VSMCs and elastic, combining into a contracting unit together. As the best-known marker for distinguishing between arteries and veins, EphrinB2 marking arteria VSMCs and the corresponding receptor ephrin type B receptor 4 (Eph-B4) marking venous VSMCs contribute a lot to neointima formation [90,91]. Protack et al. displayed increased EphrinB2 and Eph-B4 protein expressions in the outflow tract vein of human AVF and mouse aortocaval fistula [92]. Subsequently, the stimulation of Eph-B-mediated signaling showed improved fistula patency with less wall thickness through a protein kinase B (AKT)-mediated mechanism [92]. Reduced Eph-B4 expression has also been reported in human, rat, and mouse vein grafts and is not accompanied by elevated EphrinB2, leading to the thickening of the vein wall [93,94]. Meanwhile, Muto et al. found that the stimulation of Eph-B4 signaling could inhibit venous wall thickening in a mouse model of vein graft implantation, which was reversed by Eph-B4 signaling reduction, suggesting that the inhibition of the Eph-B4 signaling pathway may play an essential role in neointimal hyperplasia [94]. 

In addition to differences in origin, accumulating evidence suggests that the proliferation and migration of VSMCs via phenotypic switching are critical cellular events during neointimal formation [40,89,95]. VSMCs’ phenotypic switching is generally associated with decreased SMC contractile marker gene expressions and increased VSMC proliferation and migration [96], resulting in AVF failure. In an experiment using transgenic mice harboring a cre recombinase-dependent reporter gene, investigators found that the vast majority of cells in the neointima were derived from VSMCs in the tunica media [97]. In another experiment to determine the phenotype of SMCs in the neointima, a VSMC lineage tracing reporter mouse was used to track mature VSMCs in the AVF mouse model, and it was found that the thickened medial VSMC layer was solely composed of differentiated VSMCs, and dedifferentiated VSMCs were a major cellular component of the neointima [40]. A previous study by our group has found that mitotic arrest-deficient protein 2B (MAD2B) served as a detrimental regulator of neointimal hyperplasia, leading to the proliferation and migration of dedifferentiated VSMCs by suppressing ski-related novel gene (SnoN) degradation [98]. A recent study also showed that the inhibition of the PDGF/AKT/mammalian target of the rapamycin (mTOR) pathway with rapamycin in mouse AVF prevented intimal hyperplasia and improved AVF patency [63]. Furthermore, the inhibition of the Notch signaling pathway at 1 or 2 weeks after mouse AVF establishment maintained the expression of VSMC contractile markers, reduced neointimal formation, and promoted AVF maturation [99]. 

In summary, on the basis of clarifying the genetic background of VSMCs in different origins, how to inhibit VSMC phenotype transformation or target the subpopulation of dedifferentiated VSMCs may be an important direction to reduce neointima hyperplasia in AVF without affecting outward remodeling in the future. 

### 5.3. Myofibroblasts and Fibroblasts

Physically, fibroblasts and myofibroblasts constitute the major cellular components of tunica adventitia and mainly contribute to ECM production under different etiologic settings. Following AVF establishment, nutrient vessels in the tunica adventitia are disrupted, forming a hypoxic environment, then lead to the increased differentiation of fibroblasts into myofibroblasts and the overexpression of hypoxia-inducible factor-1α (HIF-1α) in AVF or graft failure specimens [100,101,102,103]. Another study found that selective targeting at VEGF-A expression in the adventitia of AVF outflow veins with a lentivirus-delivered small-hairpin RNA markedly reduced myofibroblast production, proliferation, and migration with decreased HIF-1α expression, ultimately preventing neointimal formation [104]. In addition, the hedgehog (HH) signaling pathway also plays a vital role in regulating the differentiation of mesenchymal stem cells into myofibroblasts, and HH overexpression induces the translocation and accumulation of PDGF receptor (PDGFR) subunit A in early endosome and then interacts with TGF-β/Smad signaling, leading to neointima formation [105]. Moreover, platelet factor 4 (PF4/CXCL4) was also found to be associated with ultimate AVF failure via a RNA-seq study, while PF4 administration could stimulate expressions of α-SMA and collagen 1 via TGF-β pathways, eventually resulting in venous fibrosis [106]. 

Therefore, as potent inducers for ECM production, fibroblasts and myofibroblasts appear essential in excessive inward remodeling after AVF establishment, significantly increasing the risk of early AVF failure, which suggests that therapeutic therapies targeting the adventitia have potential in the treatment of excessive inward remodeling-induced AVF dysfunction.

### 5.4. Inflammatory Cell Infiltration

In vascular diseases, inflammatory cell infiltration always occurs following early vascular endothelial damage and intimal matrix exposure. As for AVF stenosis, increased numbers of CD68 (macrophage marker) and CD45 (leukocyte marker)-positive cells were identified as reported, indicating inflammation infiltration [107,108], which appears significant in CKD patients under a systemic hyperinflammatory state induced by the high circulating level of uremic toxins [78]. In addition to the inflammation infiltration derived from the vascular endothelial injury, recent studies proved that inflammatory cells could also gradually infiltrate from the adventitia to the neointima. A mouse model of AVF stenosis showed that macrophage infiltration in the adventitia initiated at 2 days after AVF establishment, peaked at 7 days, and then gradually declined, which was all detected by immunohistochemical analyses. On the other hand, minimal macrophage infiltration or proliferation was found in the neointima at 7 days or 14 days after AVF creation, suggesting an outward to inward macrophage infiltration [109]. 

Furthermore, specific macrophage subtypes play different roles in regulating inward remodeling in AVF dysfunction. M1 macrophages accumulate during the early phase of AVF vascular remodeling, followed by an increase in the number of M2 macrophages during the later maturation phase. It has been suggested that the inhibition of macrophage activity with rapamycin via the AKT1-mTORC1 axis in the early stage leads to a reduction in neointimal hyperplasia and better patency, whereas the depletion of macrophages with clodronate-containing liposomes resulted in poorer remodeling and patency, suggesting a possible role of M1 macrophages in promoting neointimal hyperplasia in the early stage [63]. M1 macrophages also release various cytokines, such as TNF-α and interferon-gamma (IFN-γ), which enhance MMP upregulation and ECM remodeling, facilitating VSMC migration and promoting neointimal hyperplasia. Chen et al. confirmed that hirudin protected against vascular injury in chronic renal failure by affecting the polarization and inflammatory response of M1 macrophages [110].

Additionally, the M1 macrophage was also recognized as having an essential role in the development of atherosclerosis and fibrosis [111,112] by phagocytosing oxidized low-density lipoprotein (oxLDL), leading to the typical necrotic core lesions within the plaque [113,114]. Similarly, a retrospective study showed that elevated blood LDL and phosphorus were the major independent risk factors for disrupting AVF patency, leading to intimal hyperplasia, which suggests that M1 macrophage may also affect AVF dysfunction via lipid metabolism [115]. In addition to regulating ECM remodeling and VSMC and fibroblast migration and proliferation through inflammatory responses, macrophages can also transform into fibroblast-like cells, thus contributing to the neointima directly [116]. However, more studies are still needed to clarify the exact role of the M1 macrophage subtype during AVF stenosis.

### 5.5. Bone-Marrow-Derived Cells

In addition to the resident cells in the vessel wall, growing studies have reported that bone-marrow-derived cells also participate in neointimal hyperplasia. In a mouse model of femoral artery damage where the bone-marrow-derived cells had been replaced by that of ROSA 26 mice, which universally express β-galactosidase gene Z (LacZ), Sata et al. showed that a significant amount of LacZ^+^ cells in the neointima (63.0 ± 9.3%) and media (45.9 ± 6.9%) at one-week post-injury formed [117]. In contrast, LacZ^+^ cells were not detected in uninjured femoral arteries in wild-type mice, suggesting that bone-marrow-derived vascular progenitor cells may contribute to neointimal formation in the model of femoral artery damage [117]. Controversially, a recent study displayed that bone marrow stem cells did not contribute to SMC accumulation in neointimal lesions of AVF arteries in chimeric mice that received bone marrow implantation from transgenic mice expressing LacZ gene in smooth muscle cells (SM-LacZ) [118]. Consistently, another study adopting wild-type mice reconstituted with GFP^+^ bone marrow stem cells supported this conclusion [119]. The discrepancy between different vascular models needs to consider the fact that AVFs possess a dual identity as arterial and venous vessels, and there still needs to be great efforts to explore the nature of the role of bone-marrow-derived cells in AVF stenosis.

## 6. Outward Remodeling and Inward Remodeling: The Friends or the Foes?

It is well recognized that both insufficient outward remodeling and excessive inward remodeling exert an important effect in AVF dysfunction, including maturation failure and stenosis. Notably, outward and inward remodeling are not just two sides of a coin and have lots of characteristics in common. 

On the one hand, various types of cells including VSMCs, myofibroblasts, and inflammatory cells are involved in outward and inward remodeling collectively. For example, VSMCs contribute to vessel wall thickening by proliferating and producing ECM in outward remodeling. Similarly, VSMCs are also the major cellular component of neointima in inward remodeling. As the main regulator of ECM components, myofibroblasts mediate the thickening of the media in outward remodeling, and they also play an important role in inward remodeling by mediating excessive intimal hyperplasia [6]. Macrophages are also involved in both outward and inward remodeling by secreting a variety of cytokines. For example, macrophages secrete IL-1 to enhance outward vascular remodeling [120] and large amounts of TNF-α and IFN-γ to promote the phenotypic transformation of VSMCs, as well as promoting neointima [121,122]. Mounting studies have revealed that the inhibition of the above cells may alleviate neointima but ultimately lead to reduced AVF patency in combination with impaired outward remodeling [63,99]. 

On the other hand, outward and inward remodeling have shared diverse similar signaling pathways involved in the pathological setting. In inward remodeling, the activation of phosphatidylinositol 3-kinase (PI3K)/AKT/mTOR axis can lead to the proliferation and migration of VSMCs as well as macrophage infiltration. Eventually, this leads to AVF stenosis [63,123], while the axis has been shown to be enhanced during graft adaptation and AVF maturation [124]. Additionally, Eph-B4 receptor signaling is also reported to be mediated through AKT activation in the vein wall during AVF maturation [92]. Moreover, the Notch/RBP-Jκ signaling pathway can activate EndMT and VSMC migration, resulting in neointimal formation [86,87,89], whereas blocking the Notch/RBP-Jκ signaling pathway in VSMCs at an early stage (before or 1 week after AVF surgery) could lead to increased inflammatory cell infiltration and an un-arterialized AVF in turn [99]. Furthermore, as a classical signaling pathway, the TGF-β/Smad2/3 axis is closely involved in the proliferation and migration of VSMCs and neointimal formation [125]. On the contrary, as a non-classical signaling pathway, TGF-β/TAK1 signaling pathway activation can lead to the wall thickening and lumen diameter enlargement of AVF [41].

However, outward and inward remodeling also show various differences relying on the diverse sites, stages, and phenotypes of the involved cells. For example, inward remodeling occurs predominantly in the intima, whereas the core area of outward remodeling is always referred to as the media. However, the locations are always relative because neointimal hyperplasia alone is not statistically associated with definite AVF failure. In contrast, the combination of neointimal hyperplasia after the presence of media fibrosis dramatically increases the likelihood of AVF failure [6]. Similarly, the excessive fibrosis of the adventitia limits the outward expansion of the AVF and induces inward remodeling [105]. Additionally, different stages of occurrence can also lead to opposite outcomes for the same cellular events. It has been suggested that an early acute inflammatory response facilitates outward remodeling by favoring the reconstitution of ECM and VSMC proliferation in the tunica media [99] while chronic inflammatory response leads to persistent ECM deposition and neointimal hyperplasia, resulting in AVF failure [63,121]. Notably, differential phenotypes of cellular subtypes can also lead to a diversity of outcomes, especially VSMCs and macrophages. Explanatorily, VSMC proliferation is a common cytological event in two types of remodeling by expressing proliferative labels, such as cytokeratin-18 (CK-18), calmodulin 2 (CALM2), krüppel-like factor 4 (KLF4), and CD68; however, the dominator of VSMC phenotype in outward and inward remodeling is different due to the contractile markers [121].

Consequently, outward and inward remodeling share similarities and differences in AVF development due to various cellular events and signaling pathways, providing us with a comprehensive insight into the nature of AVF maturation and dysfunction.

## 7. The Potential Therapeutic Molecular Targets in AVF Dysfunction

**Figure 3:**
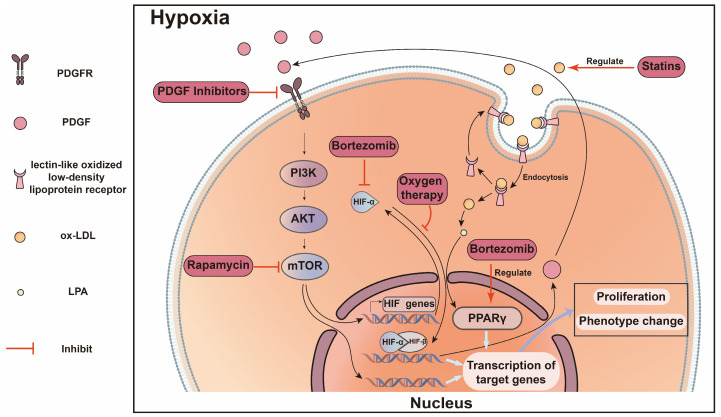
Overview of the signaling pathways involved in AVF dysfunction and potential therapeutic molecular targets. Taking VSMC as an example, after AVF surgery, PDGF secreted by ECs and other cells binds to PDGFR on the cell membrane of VSMC, which activates the intracellular PI3K/AKT/mTOR signaling pathway and leads to increased expression of genes related to proliferation, differentiation, and regulation of ECM. PDGFR inhibitor and rapamycin can inhibit PDGFR and mTOR to protect AVF. At the same time, in cells with activated PI3K/AKT/mTOR signaling pathway, the protein level and activity of HIF-α are increased. In addition, the hypoxia environment caused by AVF surgery causes HIF-α to enter the nucleus and combine with HIF-β to form a complex to obtain transcriptional activity, which binds to the hypoxia-responsive element consensus sequence on the promoter of target genes to activate the expression of genes related to cell proliferation and angiogenesis. Bortezomib and oxygen therapy inhibit HIF-α activity and nuclear translocation, thereby inhibiting neointimal formation. Vascular injury and ischemia–reperfusion injury activate the oxidative stress response, which oxidizes LDL to ox-LDL and is taken up by VSMC. LPA released by ox-LDL regulates PPAR-γ activity and thus affects the proliferation and apoptosis of VSMC. Statins and pioglitazone protect AVF, respectively, by reducing LDL levels and activating PPAR pathway levels.

From bench to bedside, the core of scientific research is providing therapeutic strategies for clinical settings. Here, we propose the following representative and translationally valuable drug identifiers or antibody-blocking molecular targets for introduction based on the abovementioned illustrations (Figure 3).

As reported, the PDGFR/PI3K/AKT/mTOR axis can regulate cell proliferation, apoptosis, and immune response physiologically, and its dysregulation has been associated with the development of various diseases, including cancer [126], diabetes [127,128], and autoimmunity, as well as AVF dysfunction [129,130]. Thus, Dasatinib, an orally bioavailable protein tyrosine kinase inhibitor currently applied in clinical cancer patients, is a potential therapeutic drug for AVF stenosis. It could function as a potent PDGFR inhibitor at low nanomolar concentrations by inhibiting VSMC proliferation and downstream PI3K/AKT activity [131]. Additionally, rapamycin could ameliorate neointimal hyperplasia by suppressing VSMC proliferation and migration and ECM deposition, as well as improve AVF patency by reducing the activation of AKT/mTORC1 and modulating the Notch pathway, clinically applied in restenosis currently, suggesting a potential therapeutic option for AVF dysfunction.

Elevated blood LDL is also a significant independent risk factor for disrupting AVF patency, leading to neointimal hyperplasia [115] and inflammatory vascular lesions. As 3-hydroxy-3-methylglutaryl-coenzyme A (HMG-CoA) reductase inhibitors, statins are an important class of drugs widely used in clinical practice to regulate blood lipids in patients. At the same time, statins can reduce thrombin generation, inhibit procoagulant reaction catalyzed by thrombin, and decrease the production of plasminogen activator inhibitor-1 [132,133], thus preventing thrombosis and fiber deposition. Moreover, treatment with statins in AVF animals increases the mean luminal vessel area by decreasing VEGF and MMP-9 gene expression, reducing fibrin deposition, and attenuating macrophage aggregation [134,135], which indicates the underlying effect of statins in clinical settings. Anti-platelet agents such as aspirin, which are obviously beneficial for thrombin generation reduction, have also widely been used in clinical settings to decrease AVF failure. However, there are some different opinions about the effects of anti-platelet agents on AVF survival among hemodialysis patients. A multicenter study enrolling 2815 incident hemodialysis patients using an AVF indicated that consistent aspirin use was significantly related to a lower risk of final AVF failure [136]. However, recent meta-analyses showed that there was insufficient evidence to determine if there was a difference in AVF patency between placebo and other treatments such as aspirin and clopidogrel [137]. Furthermore, lysophosphatidic acid (LPA), a product of the oxidative modification process of LDL [138], was produced intracellularly by lysophosphatidylcholine (LPC) via the autotaxin (ATX) or phospholipase A2 (PLA2) enzyme in a stimulus-coupled manner [139]. Recent studies have reported that LPC is highly accumulated in the media of AVF [140], and LPA can regulate cell proliferation, migration, and apoptosis by activating PPARγ as a bioactive lipid mediator and second messenger [140,141,142], providing the possibility of LPA to be applied in clinical AVF dysfunction patients. In addition, pioglitazone, a PPAR-γ agonis widely used in clinical diabetes treatments has been shown to induce PPARγ expression and down-regulate the invasion genes snail transcription factor 1 (SLUG), MMP-9, and Vimentin, thus inhibiting the proliferation and migration of ECs and VSMCs in AVF [143].

Recently, a growing number of studies have demonstrated that oxidative stress in the vessel wall plays a vital role in AVF failure under a hypoxia environment. Following AVF creation, blood supply in the outflow vein is interrupted and conversed into arterial blood with high oxygen partial pressure, leading to ischemia–reperfusion injury and oxidative stress activation. Moreover, AVF surgery cuts off small, nourishing vessels in the adventitia and makes vascular walls consistent with hypoxic coupled with intimal hyperplasia. Typically, HIF is a crucial transcription factor in the hypoxic injury response, which helps to restore oxygen and maintain efficient energy production. Meanwhile, numerous studies have shown obvious hypoxia in the vascular wall accompanied by the activation of the HIF signaling pathway in the neointima [144,145]. This suggests oxygen therapy to be the best possible treatment for AVF dysfunction by improving hypoxia. Practically, the administration of 30% supplemental oxygen inhibited intimal hyperplasia in a model of AVF in the iliac artery of rabbits [146].

Nowadays, drug-coated balloons (DCBs) related to potential therapeutic targets have been gradually developed. Paclitaxel, as a cytotoxic agent, is the most commonly used drug in drug-eluting balloons. The first DCB approved by the FDA to treat AVF stenosis was Lutonix coated with paclitaxel in 2017. Once paclitaxel coated in balloon is released, it stops the cell cycle in the M-phase of mitosis and inhibits VSMC migration in the intima, thus preventing neointimal hyperplasia [147]. A recent multicenter study recruiting 244 hemodialysis patients with fistula dysfunction displaying paclitaxel-coated balloon angioplasty achieved superior primary patency compared to plain balloon angioplasty [148]. In addition, the sirolimus-coated balloon is another type of DCB that is clinically applied in AVF dysfunction treatment due to the suppressive effect of neointimal hyperplasia via inhibiting EndMT and activating Notch signaling pathway in the ECs [86,87]. A prospective pilot study enrolling 33 ESRD patients with dysfunctional fistula suggested that sirolimus-coated balloon angioplasty for dysfunctional AVF circuits was a safe and efficacious modality in Asian hemodialysis patients at six months comparable with paclitaxel-coated balloon angioplasty [149].

Conclusively, current in vitro and in vivo research studies provide diverse therapeutic insights for us to apply molecular targets in AVF dysfunction from bench to bedside, but there still needs to be great efforts to develop more drugs and conduct clinical trials as soon as possible.

## 8. Conclusions and Perspectives

AVF dysfunction remains a significant clinical problem to be solved due to its heavy effects on longevity and life quality in hemodialysis patients. Outward remodeling and inward remodeling are currently recognized as two major parts of vascular remodeling. Meanwhile, growing studies have shown that insufficient outward remodeling and excessive inward remodeling are the pathophysiological basis of AVF dysfunction. On the one hand, outward remodeling failure is attributed to VSMC proliferation, ECM dynamic changes, and inflammatory responses. On the other hand, ECs, VSMCs, myofibroblasts, inflammatory cells, and bone-marrow-derived cells participate in excessive inward remodeling collectively through various mechanisms. Interestingly, outward and inward remodeling share many similarities in involved cell types and signaling pathways, as well as have diverse differences in the site, stage, and cell phenotype, which jointly determine the final outcome of AVF. Based on abovementioned mechanisms, we have proposed representative molecular therapeutic targets for AVF dysfunction. Therefore, we believe that it is a potential prevention and therapeutic strategy for AVF dysfunction to maintain a balance between outward and inward remodeling.
